# Re‐irradiation in patients with progressive or recurrent brain metastases from extracranial solid tumors: A novel prognostic index

**DOI:** 10.1002/cam4.4921

**Published:** 2022-06-30

**Authors:** Salvador Gutierrez Torres, Federico Maldonado Magos, Jenny G. Turcott, Juan‐Manuel Hernandez‐Martinez, Bernardo Cacho‐Díaz, Andrés F. Cardona, Aida Mota‐García, Francisco Lozano Ruiz, Maritza Ramos‐Ramirez, Oscar Arrieta

**Affiliations:** ^1^ Radiotherapy Unit Instituto Nacional de Cancerología (INCan) Mexico City Mexico; ^2^ Thoracic Oncology Unit Instituto Nacional de Cancerología (INCan) Mexico City Mexico; ^3^ Cátedras CONACYT‐Instituto Nacional de Cancerología Mexico City Mexico; ^4^ Neuro‐Oncology Unit Instituto Nacional de Cancerología (INCan) Mexico City Mexico; ^5^ Luis Carlos Sarmiento Angulo Cancer Treatment and Research Center (CTIC) Bogotá Colombia; ^6^ Foundation for Clinical and Applied Cancer Research (FICMAC) Bogotá Colombia; ^7^ Molecular Oncology and Biology Systems Research Group (FOX‐G/ONCOLGroup) Universidad El Bosque Bogotá Colombia

**Keywords:** prognostic index score, recurrent brain metastases; re‐irradiation, repeat radiotherapy

## Abstract

**Background:**

Most studies evaluating factors associated with the survival of patients with brain metastases (BM) have focused on patients with newly diagnosed BM. This study aimed to identify prognostic factors associated with survival after brain re‐irradiation in order to develop a new prognostic index.

**Methods:**

This 5‐year retrospective study included patients treated with repeat‐radiotherapy for recurrent BM at the “Instituto Nacional de Cancerología” of Mexico between 2015 and 2019. Significant variables in the multivariate Cox regression analysis were used to create the brain re‐irradiation index (BRI). Survival and group comparisons were performed using the Kaplan–Meier method and the log‐rank test.

**Results:**

Fifty‐seven patients receiving brain re‐irradiation were identified. Most patients were women (75.4%) with a mean age at BM diagnosis of 51.4 years. Lung and breast cancer were the most prevalent neoplasms (43.9% each). Independent prognostic factors for shorter survival after re‐irradiation were: Age >50 years (hazard ratio [HR]:2.5 [95% confidence interval [CI], 1.1–5.8]; *p* = 0.026), uncontrolled primary tumor (HR:5.5 [95% CI, 2.2–13.5]; *p* < 0.001), lesion size >20 mm (4.6 [95% CI, 1.7–12.2]; *p* = 0.002), and an interval <12 months between radiation treatments (HR:4.3 [95% CI, 1.7–10.6]; *p* = 0.001). Median survival (MS) after re‐irradiation was 14.6 months (95% CI, 8.2–20.9).MS of patients stratified according to the BRI score was 17.38, 10.34, and 2.82 months, with significant differences between all groups.

**Conclusions:**

The new BRI can be easily implemented for the prognostic classification of cancer patients with progressive or recurrent BM from extracranial solid tumors.

## Graphical abstract

The BRI score is a new prognostic index that can be easily implemented in clinical practice for the prognostic classification of cancer patients with progressive or recurrent brain metastases from extracranial solid tumors

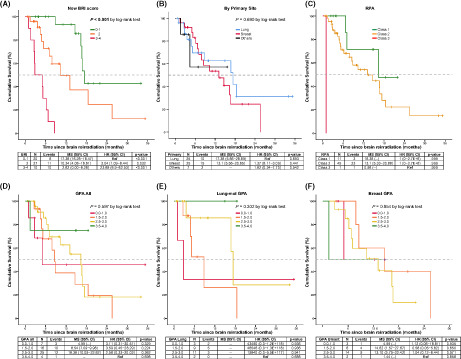

## 1 INTRODUCTION

Brain metastases (BM) are one of the most detrimental factors impacting the clinical course of cancer patients, with many of them presenting neurologic deficits that negatively affect their overall performance and quality of life.^1^ It is widely accepted that lung cancer, breast cancer, melanoma, and renal cell carcinoma have the highest incidence of BM. However, available estimates vary substantially across studies, ranging from 8.5% to over 50%.^2^, ^3^, ^4^

Advances in imaging techniques and systemic therapies have led to a higher number of early‐stage diagnoses, better progression‐free survival, and overall survival (OS), but also to a higher lifetime risk of being diagnosed with BM.^5^ The increased incidence of BM observed over the past 10 years is consistent with the low brain accumulation and activity of most anticancer drugs.^6^ Thus, surgical resection, whole‐brain radiation therapy (WBRT), and stereotactic radiosurgery (SRS), often used sequentially or in combination, have remained the preferred treatments for patients with symptomatic BM.^7^ In recent years, evidence has accumulated supporting the upfront use of systemic therapies with proven activity in the central nervous system (CNS), such as newer‐generation tyrosine kinase inhibitors and immune‐checkpoint inhibitors, in molecularly selected patients.^8^

In addition to the type of local and systemic therapies received, survival in patients with BM has been shown to be associated with other clinical factors including age, performance status, primary tumor control and histology, extracranial disease burden, as well as the number, size, volume, and location of the metastatic brain lesions.^9^, ^10^, ^11^, ^12^, ^13^ Some of these factors have been included in composite prognostic indexes such as the Disease‐Specific Graded Prognostic Assessment (DS‐GPA) scale,^14^ the Recursive Partitioning Analysis (RPA) score,^15^ the Score Index for Radio‐surgery (SIR),^16^ which can be used in clinical practice to guide treatment decisions as well as in clinical research to assess patient eligibility and to perform stratified data analysis.^17^

Although the efficacy of repeat radiotherapy, in terms of local control and survival, in patients with progressive or recurrent BM has been established,^18^ there is little agreement regarding which factors should guide patient stratification and treatment decisions.^19^ The primary objective of this study was to identify prognostic factors associated with the survival of cancer patients following a second course of radiotherapy for the treatment of progressive or recurrent BM and to develop a new prognostic index.

## 2 METHODS

### 2.1 Study design and patients

This single‐center retrospective study aimed to identify prognostic factors associated with survival after brain re‐irradiation therapy. The study included patients with brain metastases (BM) treated with a first course of local radiation therapy and who underwent a second course of radiation, due to central nervous system (CNS) recurrence or progression, at the “Instituto Nacional de Cancerología” of México (INCan) between 2015–2019. Recurrent/progressive brain metastases were defined as metastases that after initial therapy recurred/progressed anywhere in the brain (in both original and non‐original sites). Additional inclusion criteria were age (≥18 years), histopathological confirmation of cancer (extracranial solid tumor), measurable CNS disease prior to re‐irradiation, and availability of MRIs before and after treatment. Patients with cranial bone metastases or whose medical records failed to document basic clinical characteristics, treatment modality, or relevant follow‐up data were eliminated. A cohort of 57 patients fulfilled these criteria and were included in the analysis.

### 2.2 Study endpoints and assessments

The primary outcome was the median duration of survival after re‐irradiation, defined as the time between the date of re‐irradiation and the date of death (due to any cause) or last contact, right censoring data at a cutoff date for the analysis on June 31, 2020. Variables with potential prognostic value were extracted from electronic medical records at baseline, first radiation, and re‐radiation. Local responses (evaluated by brain MRIs within 2 months after each course of radiation) were determined by an independent central imaging group according to Response Evaluation Criteria in Solid Tumors version 1.1 (RECIST v1.1). The objective response rate (ORR) was defined as the proportion of patients with complete or partial responses. Disease control rate (DCR) was defined as the proportion of patients with either complete response, partial response, or stable disease. BM‐related symptoms (seizures, headaches, vertigo, nausea, as well as visual, somatic, motor, and speech deficits) were recorded at baseline and after each course of radiation, classifying symptom reduction as complete, partial, or unchanged.

Recursive Partitioning Analysis (RPA) was performed as previously described,^15^ with a class assigned to each patient for both the first and second course of brain radiation. Due to the inclusion of primary tumors without a diagnosis‐specific Graded Prognostic Assessment (GPA) index, diagnosis‐specific GPA indexes^20^, ^21^, ^22^ were calculated at the same time points for only 52 patients.

### 2.3 Statistical analysis

Data were summarized as means with standard deviations (SDs) or as medians with ranges for continuous variables, and as proportions for categorical variables. Two groups comparisons between continuous variables were performed using the Student's *t*‐test. The Chi‐square test and the Fisher's Exact Test were used for categorical data. In the very few instances where significance values differed between tests, the results from the Fisher's Exact Test were reported. Comparisons between longitudinally collected and dependent dichotomous data were performed using the McNemar's paired test, while the marginal homogeneity test was used for multi categorical dependent data. Median duration of survival after brain re‐irradiation and survival curves were estimated using the Kaplan–Meier method. Differences in the estimated survival curves were assessed, overall and between group pairs, using the log‐rank test. *p*‐values <0.05 with a two‐sided test were considered statistically significant. Univariate and multivariate Cox regression analyses were performed to identify prognostic factors significantly associated with time‐to‐event outcomes. Variables that after adjustment for potential confounders remained significant in the multivariate Cox regression analysis were used to construct a new prognostic index: The brain re‐irradiation (BRI) score. For simplicity, a score of 1 was assigned to each of these variables and score classes without significantly different survival times were grouped together. Data were analyzed using the SPSS software, package version 26 (IBM Corp.).

## 3 RESULTS

### 3.1 Population characteristics at baseline

Fifty‐seven patients with extracranial solid tumors were treated with a second course of local radiotherapy for recurrent BM between 2015–2019. Baseline clinical characteristics are shown in Table 1. Most patients were women (75.4%) with a mean age at BM diagnosis of 51.4 years (standard deviation [SD] ± 12.9 years). The most frequent neoplasms were lung and breast cancer, each found in 25 patients (≅ 43.9%). The remaining patients had a diagnosis of melanoma (*n* = 2), papillary serous ovarian cancer (*n* = 2), alveolar sarcoma (*n* = 1), uterine carcinosarcoma (*n* = 1), and non‐seminomatous germ cell tumor (*n* = 1). All patients with primary lung cancer had adenocarcinoma histology, 14 (56%) had a mutation in the epidermal growth factor receptor (*EGFR*) gene, four (16%) had rearrangements of the anaplastic lymphoma kinase (*ALK*) gene, and six (28%) did not have any common somatic mutation detected. The most common breast cancer histology was invasive ductal carcinoma, in 23 out of 25 patients (92%), followed by invasive lobular carcinoma in two patients. Molecularly, luminal A tumors (ER^+^, PR^+^, HER2^−^) were found in six (24%) patients, luminal B (ER^+^, PR^+^, HER2^+^) in seven (28%) patients, HER2 (HER2^+^, ER^−^, PR^−^) in six (24%) patients and triple negative (ER^−^, PR^−^, HER2^−^) in six (24%) patients, with a Ki67 proportion score >30% in 60% of cases and a Scarff‐Bloom‐Richardson grade (SBR) >7 in 52%.

**TABLE 1:** Baseline characteristics of patients with brain metastases

Variable	Total = 57
	*n* (%)
Age (years)	
Mean ± SD	51.4 ± 12.9
Median (IQR)	50 (42–60.5)
Sex	
Men	14 (24.6)
Women	43 (75.4)
Primary tumor site	
Lung	25 (43.9)
Breast	25 (43.9)
Melanoma	2 (3.5)
Other	5 (8.7)
Histology of primary tumor	
Adenocarcinoma	25 (43.9)
Invasive ductal	23 (40.4)
Alveolar sarcoma	1 (1.8)
Carcinosarcoma	1 (1.8)
Testicular embryonal carcinoma	1 (1.8)
Cutaneous melanoma	2 (3.5)
Ovarian papillary serous	2 (3.5)
Invasive lobular	2 (3.5)
Mutations (lung cancer)	
None	7 (28)
EGFR	14 (56)
ALK	4 (16)
Molecular subtype (breast cancer)	
Luminal A	6 (24)
Luminal B	7 (28)
HER2+	6 (24)
Triple negative	6 (24)
Ki‐67 (breast cancer)	
≤30	10 (40)
>30	15 (60)
SBR (breast cancer)	
≤7	12 (48)
>7	13 (52)

Abbreviations: ALK, anaplastic lymphoma kinase; EGFR, epidermal growth factor receptor; HER2, human epidermal growth factor receptor 2; IQR, Interquartile range; SBR, Scarff‐Bloom‐Richardson grade; SD, standard deviation.

### 3.2 Population characteristics at first and second irradiation

**FIGURE 1:**
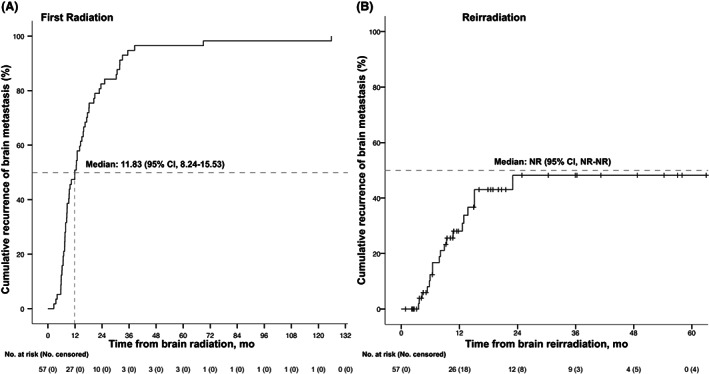
Brain metastases progression and recurrence rates. (A) Rate of brain metastasis recurrence in 57 after a first course of brain radiation and after re‐irradiation (B). Dashed lines indicate the proportion of patients with recurrence to the central nervous system at 12 and 24 months

The characteristics of patients at first radiation and re‐irradiation are shown in Table 2. The median time between radiotherapy courses was 13.4 months (interquartile range [IQR], 7.9–19.7 months). The recurrence rate of BM after re‐irradiation was 29% at 12 months, and 48% at 24 months (Figure 1). At the time of the first radiotherapy, 42 (73.7%) patients had an Eastern Cooperative Oncology Group performance status (ECOG‐PS) between 0 and 1, and 15 (26.3%) between 2 and 3. In contrast, at re‐irradiation 31 (54.4%) patients had an ECOG‐PS of 0–1 and 26 (45.6%) an ECOG‐PS of 2–3, a change that was statistically significant in a paired analysis (*p* = 0.013). The proportion of patients achieving primary tumor control increased from 38.6% (*n* = 22), at the first radiation, to 54.4% (*n* = 31) at re‐irradiation (*p* = 0.049). Regarding radiation techniques, while the percentage of patients receiving WBRT decreased from 87.6% to 56.2% between the first and the second course of radiotherapy, the percentage of patients receiving conformal radiotherapy increased from 3.6% to 35.1% (*p* < 0.001). Similarly, there was a statistically significant reduction in the radiation dose (*p* < 0.001) and in the number of fractions (*p* < 0.001), with hypo‐fractionated schemes predominating at re‐irradiation. In contrast, there were no significant differences between radiotherapy courses in terms of KPS, GPA, RPA, or extracranial metastatic involvement.

**TABLE 2:** Characteristics of patients at first and second radiotherapy

Variable	First radiation	Re‐irradiation	*p*
	*n* (%)	*n* (%)	
ECOG‐PS			
0–1	42 (73.7)	31 (54.4)	**0.013**
2–3	15 (26.3)	26 (45.6)	
KPS			
<80	10 (17.5)	15 (26.3)	0.267
≥80	47 (82.5)	42 (73.7)	
GPA index			
0.0–1.0	6 (10.5)	7 (12.3)	0.102
1.5–2.0	18 (31.6)	16 (28.1)	
2.5–3.0	16 (28.1)	25 (43.9)	
3.0–4.0	12 (21.1)	4 (7)	
RPA class			
I	12 (21.1)	11 (19.3)	0.999
II	43 (75.4)	45 (78.9)	
III	2 (3.5)	1 (1.8)	
Primary tumor status			
Controlled	22 (38.6)	31 (54.4)	**0.049**
Uncontrolled	35 (61.4)	26 (45.6)	
Extracranial metastases			
Absent	21 (36.8)	20 (35.1)	0.999
Present	36 (63.2)	37 (64.9)	
No. of brain metastases			
1	15 (26.3)	11 (19.3)	0.233
2	10 (17.5)	15 (26.3)	
≥3	32 (56.1)	31 (54.4)	
Largest lesion size (mm)			
Mean ± SD	20.7 ± 11.0	18.7 ± 10.1	0.166
Lesion location			
Supratentorial	32 (56.1)	24 (42.1)	0.198
Infratentorial	6 (10.5)	9 (15.8)	
Both	19 (33.3)	24 (42.1)	
Local therapy			
WBRT	50 (87.6)	32 (56.2)	**<0.001**
SRS	5 (8.8)	5 (8.8)	
CRT	2 (3.6)	20 (35.1)	
Radiation dose (Gy)			
<30	9 (15.8)	49 (86)	**<0.001**
≥30	48 (84.2)	8 (14)	
No. of fractions			
1	5 (8.8)	9 (15.8)	**0.001**
5	3 (5.3)	22 (38.6)	
≥10	49 (86)	26 (45.6)	
Neurological deficits			
Absent	18 (31.6)	14 (24.6)	0.503
Present	39 (68.4)	43 (75.4)	
Headache	27 (47.4)	26 (45.6)	0.999
Vertigo	15 (26.3)	17 (29.8)	0.824
Sensory	3 (5.3)	4 (7)	0.999
Motor	10 (17.5)	16 (28.1)	0.146
Nausea	11 (19.3)	6 (10.5)	0.302
Seizures	2 (3.5)	5 (8.8)	0.453
Visual	5 (8.8)	3 (5.3)	0.727
Speech	1 (1.8)	4 (7)	0.375
Neurological response			
Complete resolution	31 (54.4)	25 (43.9)	0.210
Partial improvement	6 (10.5)	18 (31.6)	
No improvement	2 (3.5)	0 (0)	
Local response			
Complete response	4 (7.0)	0 (0)	0.687
Partial response	25 (43.9)	14 (24.6)	
Stable disease	20 (35.1)	26 (45.6)	
Progressive disease	8 (14.0)	8 (14)	
Not available	0 (0)	9 (15.8)	

Abbreviations: CRT, conformal radiotherapy; ECOG‐PS, Eastern Cooperative Oncology Group Performance Status; GPA, graded prognostic assessment; KPS; Karnofsky performance status; RPA, recursive partitioning analysis; SD, standard deviation; SRS, stereotactic radiosurgery; WBRT, whole‐brain radiotherapy.

Statistically significant values (*p* < 0.05) are evidenced in bold.

### 3.3 Neurological symptoms and response

BM‐related neurological deficits and responses after the first and second radiation are shown in Table 2. Whereas 18 patients reported being asymptomatic prior to the start of the first radiation treatment, 14 reported being asymptomatic prior to the beginning of the second cycle of brain radiation. The most frequent focal neurological deficits reported by patients prior to either the first course of radiation or the second course of radiation were headache (47.4% and 45.6%), vertigo (26.3% and 29.8%), nausea (19.3% and 10.5%), and motor impairment (17.5 vs. 28.1%). After the first radiation, 31 (54.4%) patients reported complete symptom resolution, six (10.5%) patients reported partial symptom improvement, and the remaining two (3.5%) patients reported no improvement. In contrast, after brain re‐irradiation 25 (43.9%) patients reported complete symptom resolution and 18 (31.6%) patients reported a partial improvement. The only factor significantly associated with a favorable neurologic response was age at re‐irradiation (Table 3). In contrast to patients with partial symptom improvement, patients with complete symptom resolution were more likely to be ≤50 years old (*p* = 0.006).

**TABLE 3:** Factors associated with favorable neurologic responses to re‐irradiation

Variable	Complete resolution	Partial improvement	*p*
	Total = 25	Total = 18	
	*n* (%)	*n* (%)	
Age (years)			
≤50	18 (72)	5 (27.8)	**0.006**
>50	7 (28)	13 (72.2)	
ECOG‐PS			
0–1	11 (44)	8 (44.4)	0.999
2–3	14 (56)	10 (55.6)	
KPS			
<80	6 (24)	8 (44.4)	0.198
≥80	19 (76)	10 (55.6)	
GPA index			
0–2	9 (36)	11 (61.1)	0.341
>2	13 (52)	7 (38.9)	
RPA class			
I	5 (20)	1 (5.6)	0.224
II	20 (80)	17 (94.4)	
Primary tumor status			
Controlled	16 (64)	7 (38.9)	0.130
Uncontrolled	9 (36)	11 (61.1)	
Extracranial metastases			
Absent	11 (44)	3 (16.7)	0.099
Present	14 (56)	15 (83.3)	
Mutations (lung cancer)			
None	2 (15.3)	4 (66.7)	0.107
*EGFR*	7 (53.8)	2 (33.3)	
*ALK*	4 (30.7)	0 (0)	
Molecular subtype (breast cancer)			
Luminal A	3 (33.3)	1 (10)	0.760
Luminal B	2 (22.2)	2 (20)	
HER2+	2 (22.2)	3 (30)	
Triple negative	2 (22.2)	4 (40)	
Ki‐67 (breast cancer)			
≤30	3 (33.3)	3 (30)	0.999
>30	6 (66.7)	7 (70)	
SBR (breast cancer)			
≤7	3 (33.3)	4 (40)	0.999
>7	6 (66.7)	6 (60)	
No. Brain metastases at reirradiation			
1	4 (16)	4 (22.2)	0.204
2	6 (24)	3 (16.7)	
≥3	15 (60)	11 (61.1)	
Largest lesion size (mm)			
≤20	17 (68)	8 (44.4)	0.210
>20	8 (32)	10 (55.6)	
Lesion location			
Supratentorial	11 (44)	7 (38.9)	0.831
Infratentorial	4 (16)	2 (11.1)	
Both	10 (40)	9 (50)	
Local therapy			
WBRT	15 (60)	9 (50)	0.550
SRS	2 (8)	0 (0)	
CRT	8 (32)	9 (50)	
Radiation dose (Gy)			
<30	21 (84)	15 (83.3)	0.999
≥30	4 (16)	3 (16.7)	
No. of fractions			
1	5 (20)	1 (5.6)	0.153
5	7 (28)	10 (55.6)	
10	13 (52)	7 (38.9)	

Abbreviations: *ALK*, anaplastic lymphoma kinase; CRT, conformal radiotherapy; ECOG‐PS, Eastern Cooperative Oncology Group Performance Status, *EGFR*, epidermal growth factor receptor; HER2, human epidermal growth factor receptor 2; GPA, graded prognostic assessment; KPS; Karnofsky performance status; RPA, recursive partitioning analysis; SBR, Scarff‐Bloom‐Richardson grade; SRS, stereotactic radiosurgery; WBRT, whole‐brain radiotherapy.

Statistically significant values (*p* < 0.05) are evidenced in bold.

### 3.4 Radiological response

Local tumor responses after the first and second course of radiation are shown in Table 2. After initial radiotherapy, four patients (7%) had a complete response, 25 (43.9%) a partial response. Stable disease was reported in 20 patients (35.1) and disease progression in eight patients (14%). After re‐irradiation, 14 patients (29.2%) had a partial response and 26 patients (54.2%) had stable disease. There were no re‐irradiated patients with a complete response.

Response rates according to primary tumor diagnosis are shown in Table 4. After the first radiation, complete responses were observed only among breast cancer patients (4 out of 25). Similarly, partial responses were more frequent among breast cancer patients than in patients with other malignancies, who were more likely to have stable or progressive disease (*p* = 0.049). The objective response rate for the entire population was higher after the first radiation than after re‐irradiation (50.9% vs. 29.2%), with up to 72% of breast cancer patients achieving an objective response (*p* = 0.018). In contrast, there were no significant differences between malignancies with regards to the proportion of patients with specific responses after re‐irradiation. Of note, the probability of achieving an objective response after re‐irradiation was lower among breast cancer patients who had responded to the first course of radiation (*p* = 0.007).

**TABLE 4:** Local response to first and second brain radiation

Tumor Response	All	Lung cancer	Breast cancer	Other	*p*
First radiation	*N* = 57	*n* = 25	*n* = 25	*n* = 7	
Complete response	4 (7)	0 (0)	4 (16)	0 (0)	**0.049**
Partial response	25 (43.9)	9 (36)	14 (56)	2 (28.6)	
Stable disease	20 (35.1)	13 (52)	4 (16)	3 (42.9)	
Progressive disease	8 (14)	3 (12)	3 (12)	2 (28.6)	
ORR	29 (50.9)	9 (36)	18 (72)	2 (28.6)	**0.018**
DCR	49 (86)	22 (88)	22 (88)	5 (71.4)	0.497

Second radiation	*n* = 48	*n* = 18	*n* = 24	*n* = 6	
Complete response	0 (0)	0 (0)	0 (0)	0 (0)	0.454
Partial response	14 (29.2)	7 (38.8)	6 (25)	1 (16.7)	
Stable disease	26 (54.2)	10 (55.6)	13 (54.2)	3 (50)	
Progressive disease	8 (16.6)	1 (5.6)	5 (20.8)	2 (33.3)	
ORR	14 (29.2)	7 (38.9)	6 (25)	1 (16.7)	0.477
DCR	40 (83.3)	17 (94.4)	19 (79.2)	4 (66.7)	0.212
First vs. second radiation					
ORR^a^		0.999^a^	**0.007** ^a^	0.999^a^	
DCR^a^		0.999^a^	0.625^a^	0.999^a^	

Abbreviations: DCR, disease control rate; ORR, objective response rate.

Statistically significant values (*p* < 0.05) are evidenced in bold.

^a^ McNemar's test *p*‐value.

### 3.5 Survival

Table 5 shows the median survival of patients after re‐irradiation and the Cox regression analyses. At the time of data cutoff, median overall survival for the entire cohort was 59.56 months (95% confidence interval [CI], 38.508–80.621) and median survival after the first brain radiation was 35.77 (95% CI, 29.08–42.47). Median survival after brain re‐irradiation was 14.6 months (95% CI, 8.2–20.9) with survival duration for the entire population ranging from 0.69 months to 35.22 months. In the univariate analysis, variables significantly associated with the hazard ratio (HR) of death were: Age >50 years (HR: 2.1 [95% CI, 1.0–4.7]; *p* = 0.048), lesion size >20 mm (HR: 2.6 [95% CI, 1.1–6.3]; *p* = 0.026), <12 months between radiation treatments (HR: 0.3 [95% CI 0.1–0.8]; *p* = 0.012), uncontrolled primary tumor at re‐irradiation (HR: 2.1 [95% CI, 1.0–4.6]; *p* = 0.045), and post‐treatment presence of neurological symptoms (HR: 3.5 [95% CI, 1.6–7.5]; *p* = 0.001). Variables that remained significant after adjustment were: Age >50 years (HR:2.5 [95% CI, 1.1–5.8]; *p* = 0.026), uncontrolled primary tumor (HR:5.5 [95% CI, 2.2–13.5]; *p* < 0.001), lesion size >20 mm (4.6 [95% CI, 1.7–12.2]; *p* = 0.002), and time <12 months between radiation treatments (HR:4.3 [95% CI, 1.7–10.6]; *p* = 0.001).

**TABLE 5:** Factors associated with survival after re‐irradiation

Variable	N (Deaths)	Median (CI 95%)	*p*	Univariate	*p*	Multivariate	*p*
				HR (CI 95%)		HR (CI 95%)	
Survival after reirradiation							
Overall	57 (27)	14.6 (8.2–20.9)					
Age (years)							
≤50	29 (11)	16.6 (13.7–19.5)	**0.043**	Ref		Ref	
>50	28 (16)	8.5 (1.8–15.2)		2.1 (1.0–4.7)	**0.048**	2.5 (1.1–5.8)	**0.026**
ECOG‐PS							
0–1	31 (13)	16.3 (11.9–20.8)	0.475				
2–3	26 (14)	10.3 (1.4–19.2)					
KPS							
<80	15 (10)	8.5 (3.6–13.4)	0.186				
≥80	42 (17)	16.3 (13.3–19.3)					
GPA index							
≤2	23 (13)	8.5 (4.2–12.8)	0.301				
>2	29 (13)	16.6 (11.4–21.7)					
RPA class							
I	11 (3)	16.3 (NR)	0.178				
II‐III	46 (24)	13.1 (5.2–20.9)					
Primary tumor status							
Controlled	31 (12)	16.6 (16.0–17.2)	**0.040**	Ref		Ref	
Uncontrolled	26 (15)	8.8 (1.9–15.6)		2.1 (1.0–4.6)	**0.045**	5.5 (2.2–13.5)	**<0.001**
Extracranial metastases							
Absent	20 (7)	16.7 (8.9–24.5)	0.132				
Present	37 (20)	13.1 (5.3–20.8)					
Mutations (lung cancer)							
None	7 (4)	5.1 (2.3–7.9)	0.288				
*EGFR*	14 (4)	17.3 (16.1–18.6)					
*ALK*	4 (2)	8.8 (0.3–17.2)					
Molecular subtype (breast cancer)							
Luminal A	6 (2)	14.6 (5.5–23.7)	0.387				
Luminal B	7 (5)	7.0 (4.7–9.3)					
HER2+	6 (4)	10.3 (2.8–17.8)					
Triple negative	6 (4)	16.6 (0–35.4)					
Ki‐67 (breast cancer)							
≤30	10 (5)	14.6 (6.2–23.0)	0.391				
>30	15 (10)	10.3 (6.3–14.3)					
SBR (breast cancer)							
≤7	12 (7)	13.1 (0.4–25.7)	0.923				
>7	13 (8)	14.6 (5.4–23.7)					
Neurologic symptoms							
Absent	18 (15)	16.7 (15.2–18.2)	**0.001**	Ref			
Present	39 (12)	6.1 (3.5–8.7)		3.5 (1.6–7.5)	**0.001**		
No. Brain metastases							
≤3	31 (14)	16.3 (13.4–19.2)	0.587				
>3	26 (13)	10.3 (7.0–13.5)					
Largest lesion size (mm)							
≤20	34 (15)	16.7 (13.3–20.1)	**0.021**	Ref		Ref	
>20	23 (12)	7.0 (4.1–9.9)		2.6 (1.1–6.3)	**0.026**	4.6 (1.7–12.2)	**0.002**
Lesion location							
Supratentorial	24 (11)	16.3 (12.0–20.6)	0.307				
Infratentorial	9 (5)	25.5 (2.6–48.4)					
Both	24 (11)	13.1 (5.6–20.5)					
Local therapy							
WBRT	32 (16)	NR	0.066				
SRS	5 (0)	NR					
CRT	20 (11)	NR					
Radiation dose (Gy)							
<30	49 (24)	14.6 (4.5–24.6)	0.366				
≥30	8 (3)	17.3 (10.8–23.9)					
No. of Fractions							
1	9 (2)	NR	**0.033**	1.0 (0.9–1.1)	0.437		
5	22 (15)	8.5 (5.6–11.3)					
10	26 (10)	17.3 (6.4–28.2)					
Time between RT							
<12 months	25 (17)	7.0 (4.7–9.3)	**0.009**	Ref		4.3 (1.7–10.6)	**0.001**
≥12 months	32 (10)	16.7 (15.3–18.1)		0.3 (0.1–0.8)	**0.012**	Ref	

Abbreviations: *ALK*, anaplastic lymphoma kinase; CRT, conformal radiotherapy; ECOG‐PS, Eastern Cooperative Oncology Group Performance Status, *EGFR*, epidermal growth factor receptor; HER2, human epidermal growth factor receptor 2; GPA, graded prognostic assessment; KPS; Karnofsky performance status; RPA, recursive partitioning analysis; SBR, Scarff‐Bloom‐Richardson grade; SRS, stereotactic radiosurgery; WBRT, whole‐brain radiotherapy.

Statistically significant values (*p* < 0.05) are evidenced in bold.

### 3.6 Brain re‐irradiation index (BRI)

**FIGURE 2:**
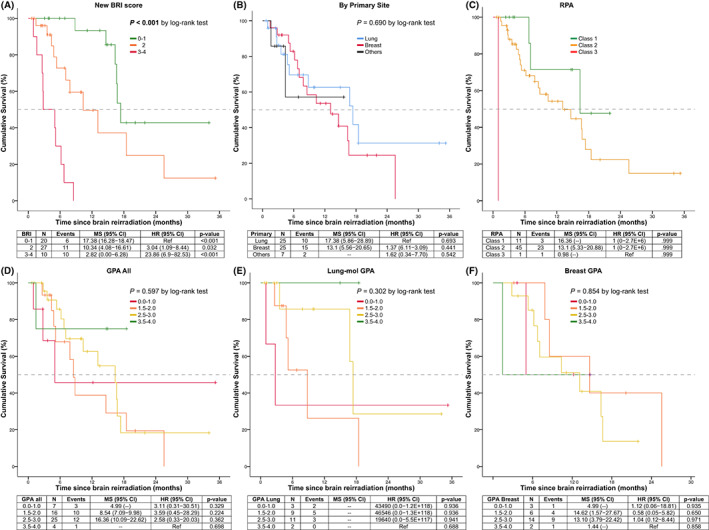
Survival after repeat radiation. Kaplan–Meier curve showing the median survival after repeat radiation (in months) of 57 patients classified according to (A) the new BRI score; (B) primary tumor site; (C) the RPA score; (D) Pooled DS‐GPA (*n* = 52); (E) The Lung‐molecular GPA score for Non–Small‐Cell Lung Cancer (*n* = 25); (F) the breast cancer GPA (*n* = 25)

Since each of the four variables that remained significant in the multivariate analysis had similar effect sizes, a score of 1 was assigned to age (>50 years), primary tumor status (uncontrolled), BM size (>20 mm), and time between radiotherapies (<12 months) to create a 3‐tiered prognostic index (scores of 0–1, 2, and 3–4), with the highest score corresponding to the highest risk of death. Of 57 patients, 20 (35%) had a score of 0–1, 27 (47.4%) had a score of 2, and 10 (17.6%) had a score of 3–4. The median survival after re‐irradiation of patients classified according to the BRI score were 17.38 month (95% CI, 16.2–18.4) for the score of 0–1, 10.34 months (95% CI, 4.0–16.6) for the score of 2, and 2.82 months (95% CI, 0.0–6.2) for the score 3–4, with significant survival differences across all groups (*p* = <0.001) and between pairs (Figure 2A). In contrast, there was no statistical difference in the duration of survival after re‐irradiation according to primary diagnosis (Figure 2B). When patients were classified according to the recursive partitioning analysis (RPA) score (Figure 2C), significant survival differences were found across groups (*p* < 0.001). However, when pairwise comparisons were performed, only Classes 1 and 2 differed from Class 3 (*p* = 0.001), with no differences between Group 1 and group 2 (*p* = 0.202). Of note, at the time of re‐irradiation only one patient with a KPS below 70 was assigned to the RPA Class 3 (Table 2). Similarly, no survival differences were found when patients were classified according to the DS‐GPA, either in the pooled analysis (Figure 2D), or when lung cancer (Figure 2E) and breast cancer were analyzed separately (Figure 2F), with *p*‐values of 0.597, 0.302, and 0.854, respectively.

## 4 DISCUSSION

Several groups have put forward prognostic factors^9^, ^10^, ^11^, ^12^, ^13^ and indexes^14^, ^15^, ^16^, ^17^, ^20^, ^21^, ^22^ for patients with newly diagnosed BM, some of which have been independently validated and increasingly being used in clinical practice and research. In contrast, clinical decisions in patients with recurrent BM are highly individualized and remain largely dependent on the clinical judgment of oncologists.

Notwithstanding, a number of studies have identified predictors of longer survival in patients treated with repeat‐SRS^18^, ^19^, ^23^, ^24^, ^25^, ^26^, ^27^ or repeat‐WBRT,^19^, ^28^, ^29^, ^30^, ^31^, ^32^, ^33^, ^34^, ^35^, ^36^ albeit with conflicting findings. This study produced results which corroborate that age (≥50 years old),^25^ uncontrolled primary tumor status,^32^, ^34^, ^36^ larger BM volume,^37^, ^38^ and shorter intervals between radiation treatments (<12 months)^24^, ^33^, ^35^, ^36^ are independent prognostic factors associated with shorter median survival (MS) after re‐irradiation. Based on these factors a new prognostic index was created, the brain re‐irradiation index (BRI), which classified patients into three risk groups with significantly different MS after re‐irradiation.

It has been reported that prognostic factors differ between patients with newly diagnosed BM and patients with recurrent BM.^9^, ^25^ However, a limited number of studies have evaluated whether prognostic indexes developed in patients with newly diagnosed BM can be used in patients with recurrent BM.^36^, ^39^, ^40^ Thus, for comparison purposes, survival was analyzed using two commonly used indexes, the Diagnostic‐Specific Graded Prognostic Assessment (DS‐GPA) for melanoma, breast and lung cancer,^20^, ^21^, ^22^ and the recursive partitioning analysis (RPA).^15^ Of note, balanced group sizes could only be obtained with the new BRI score. Furthermore, in five patients with primary tumors for which there is currently no DS‐GPA, prognosis was successfully assessed using the BRI score.

The DS‐GPA index failed to classify patients into groups with significantly different risks of death after re‐irradiation. This result may be explained by the fact that the DS‐GPA was developed to estimate the survival of patients with newly diagnosed BM. Thus, excluded from the analysis were patients with recurrent BM or leptomeningeal metastases, which are the most frequently diagnosed metastases in long‐term survivors. Another possible explanation is that the DS‐GPA for each patient was calculated using the mutation status results from diagnostic tumor biopsies, since updating this information at re‐irradiation would have required obtaining repeat tissue biopsies. In light of the well‐known limitations of tissue biopsies and the growing use of liquid biopsies for the detection of clinically actionable mutations,^41^ it will be important to determine whether mutation status results from liquid biopsies can be integrated into existing prognostic indexes or used for the development of novel prognostic systems. It has been noted that the DS‐GPA scale can be used to calculate the life expectancy of patients at any given time point after treatment initiation.^14^, ^42^ However, this approach might not be suitable for patients with recurrent BM who have different survival trajectories.

The results of this study stand in contrast with previous research showing that the presence neurological symptoms^30^, ^33^, ^35^ and a larger number of BM^25^, ^39^ are independent predictors of reduced survival. However, they are consistent with other studies in which survival has not been found to be associated with neurological symptoms^34^, ^36^, ^43^ or with the number of BM.^9^, ^24^, ^27^

In contrast to other studies,^31^, ^33^, ^34^, ^35^, ^36^ Kased et al., previously reported that KPS (≥70) was an independent prognostic factor of longer survival in newly diagnosed patients but not in patients treated with repeat SRS for recurrent BM.^9^ In the current study, KPS was not found to be a significant predictor of survival after re‐irradiation in either the univariate or the multivariate analysis. A possible explanation for this might be the underrepresentation of patients with a low KPS, which could be the result of selection and information bias. For instance, patients with a low KPS are less likely to be treated with repeat radiotherapy due to death, loss‐to‐follow‐up, or lack of consent. Similarly, patients with a low KPS score are more frequently offered best supportive care rather than repeat radiotherapy, which according to most clinical recommendations is reserved for patients with good prognosis. Finally, given the subjectivity of KPS scoring, the possibility of assessment bias cannot be excluded. This accords with a study by Caballero et al., in which the prognostic value of KPS could not be assessed because of a limited number of patients with low scores.^25^ More recently, it has been noted that inclusion criteria based on KPS is too restrictive, even in studies of patients with poor prognosis.^42^ Similarly, it has been noted that the selection of variables to be included in a multivariate model requires careful consideration since, for instance, the RPA and DS‐GPA overlap and are not independent of age, KPS and extracranial disease status.^44^ In this study, the survival analysis of patients stratified according to RPA scores did not yield meaningful results as it has in previous studies.^16^, ^23^, ^36^

Importantly, whereas the population of this study included patients treated between 2015 and 2019, previous studies had been restricted to the analysis of patients treated up to the year 2011,^28^, ^29^, ^30^, ^33^, ^34^, ^36^, ^45^ and only two studies included patients treated up to 2014.^27^, ^35^ During this time there have been important advances in the development of systemic and local therapies, imaging techniques, molecular profiling, etc., which together have improved the clinical outcomes of cancer patients. Historic series have reported MS times after repeat WBRT ranging from 2.0 to 5.3 months.^19^ Since in this study a large proportion of patients were treated with repeat‐SRS, the MS estimates herein reported are broadly consistent with other studies of repeat‐SRS, in which MS ranges from 3.0 to 10.8 months.^19^ Although the impact of targeted therapies was not evaluated, its contribution to improved MS or its possible association with the higher KPS scores observed cannot be excluded.

The lack of association between KPS and survival precluded the possibility of performing an exhaustive evaluation of other indexes, which rely on the performance status of patients,^17^ including the re‐irradiation score for patients treated with repeat‐WBRT proposed by Logie et al. in 2017.^36^ Nevertheless, what is clear from our results is that even in a population where most patients had a good KPS (≥80), there are marked survival differences that are related to other factors, further highlighting the importance of developing composite indexes specific for patients with recurrent BM as well as the urgent need to incorporate them into routine clinical practice.

## 5 CONCLUSIONS

The proposed BRI can be easily implemented in clinical practice for the prognostic classification of cancer patients with progressive or recurrent BM arising from extracranial solid tumors and could potentially be used to help guide therapeutic decisions after a first course of local radiotherapy. The BRI has the added advantage of using clinical variables that are less prone to subjective interpretation. Nonetheless, considering the relatively small sample size and the retrospective nature of this study, prospective and larger‐scale studies are necessary to validate this novel prognostic index.

## AUTHOR CONTRIBUTIONS

Salvador Gutierrez Torres: Project conceptualization, cases collection, data curation and investigation, methodology, data interpretation and formal analysis, data visualization, original draft writing, draft review and editing. Federico Maldonado Magos: Project conceptualization, data curation and investigation, methodology, data interpretation and formal analysis, data visualization, project administration and supervision, original draft writing, draft review and editing. Jenny G Turcott: Project conceptualization, data curation and investigation, methodology, data interpretation and formal analysis, data visualization, project administration and supervision, original draft writing, draft review and editing. Juan‐Manuel Hernandez‐Martinez: data curation and investigation, methodology, data interpretation and formal analysis, data visualization, original draft writing, draft review and editing. Bernardo Cacho‐Díaz: Cases collection and methodology, draft review and editing. Andrés F. Cardona: Cases collection and methodology, draft review and editing. Aida Mota‐García: Cases collection and methodology, draft review and editing. Francisco Lozano Ruiz: Cases collection and methodology, draft review and editing. Maritza Ramos‐Ramirez: Cases collection, draft review and editing. Oscar Arrieta: Project conceptualization, data curation, data interpretation and formal analysis, funding acquisition, methodology, project administration and supervision, and writing‐review and editing. All authors read and approved the final manuscript.

## FUNDING INFORMATION

This study did not receive any specific funding from agencies in the public, commercial, pharmaceutical, or from the for‐non‐profit sector.

## CONFLICT OF INTERESTS

OA reports personal fees from Pfizer, grants and personal fees from Astra Zeneca, grants and personal fees from Boehringer Ingelheim, personal fees from Lilly, personal fees from Merck, personal fees from Bristol Myers Squibb, grants and personal fees from Roche; all of them outside the submitted work; Dr Hernandez‐Martinez has received grants, awards and personal fees from CONACyT. Cardona reported receiving grants or personal fees from Roche, Boehringer Ingelheim, AstraZeneca, Pfizer, Celldex, Bristol‐Myers Squibb, Merck Sharp & Dohme, and AbbVie and reported being cofounder of the Foundation for Clinical and Applied Cancer Research (FICMAC), Bogotá, Colombia. No other disclosures were reported.

## ETHICS STATEMENT

This study was approved by the Institutional Review Board and Ethics Committee of INCan [(INCAN (2020/0025)] and conducted in accordance with the Code of Ethics of the World Medical Association (Declaration of Helsinki), following local laws and all applicable regulatory requirements on observational studies.

## INFORMED CONSENT

This was a retrospective study that used de‐identified registries. Thus, the Institutional Review Board and Ethics Committee of INCan waived the requirement of obtaining formal informed consent.

## Data Availability

All data generated or analyzed during this study are included in this published article. Original datasets are available from the corresponding author on reasonable request.

## References

[1] Achrol AS , Rennert RC , Anders C , et al. Brain Metastases. Nat Rev Dis Primers. 2019;5:5. 30655533 10.1038/s41572-018-0055-y

[2] Schouten LJ , Rutten J , Huveneers HA , Twijnstra A . Incidence of brain metastases in a cohort of patients with carcinoma of the breast, colon, kidney, and lung and melanoma. Cancer. 2002;94:2698‐2705. 12173339 10.1002/cncr.10541

[3] Barnholtz‐Sloan JS , Sloan AE , Davis FG , Vigneau FD , Lai P , Sawaya RE . Incidence proportions of brain metastases in patients diagnosed (1973 to 2001) in the metropolitan Detroit cancer surveillance system. J Clin Oncol. 2004;22:2865‐2872. 15254054 10.1200/JCO.2004.12.149

[4] Nayak L , Lee EQ , Wen PY . Epidemiology of brain metastases. Curr Oncol Rep. 2012;14:48‐54. 22012633 10.1007/s11912-011-0203-y

[5] Thapa B , Lauko A , Desai K , Venur VA , Ahluwalia MS . Novel systemic treatments for brain metastases from lung cancer. Curr Treat Options Neurol. 2018;20:48. 30259236 10.1007/s11940-018-0533-2

[6] Angeli E , Nguyen TT , Janin A , Bousquet G . How to make anticancer drugs cross the blood‐brain barrier to treat brain metastases. Int J Mol Sci. 2019;21:21. 31861465 10.3390/ijms21010022 PMC6981899

[7] Suh JH , Kotecha R , Chao ST , Ahluwalia MS , Sahgal A , Chang EL . Current approaches to the management of brain metastases. Nat Rev Clin Oncol. 2020;17:279‐299. 32080373 10.1038/s41571-019-0320-3

[8] Soffietti R , Ahluwalia M , Lin N , Ruda R . Management of brain metastases according to molecular subtypes. Nat Rev Neurol. 2020;16:557‐574. 32873927 10.1038/s41582-020-0391-x

[9] Kased N , Binder DK , McDermott MW , et al. Gamma knife radiosurgery for brain metastases from primary breast cancer. Int J Radiat Oncol Biol Phys. 2009;75:1132‐1140. 19345514 10.1016/j.ijrobp.2008.12.031

[10] Karlsson B , Hanssens P , Wolff R , Soderman M , Lindquist C , Beute G . Thirty years' experience with gamma knife surgery for metastases to the brain. J Neurosurg. 2009;111:449‐457. 19199505 10.3171/2008.10.JNS08214

[11] Hunter GK , Suh JH , Reuther AM , et al. Treatment of five or more brain metastases with stereotactic radiosurgery. Int J Radiat Oncol Biol Phys. 2012;83:1394‐1398. 22209150 10.1016/j.ijrobp.2011.10.026

[12] Banfill KE , Bownes PJ , St Clair SE , Loughrey C , Hatfield P . Stereotactic radiosurgery for the treatment of brain metastases: impact of cerebral disease burden on survival. Br J Neurosurg. 2012;26:674‐678. 22747250 10.3109/02688697.2012.690913

[13] Balasubramanian SK , Sharma M , Venur VA , et al. Impact of EGFR mutation and ALK rearrangement on the outcomes of non‐small cell lung cancer patients with brain metastasis. Neuro Oncol. 2020;22:267‐277. 31648302 10.1093/neuonc/noz155 PMC7442419

[14] Sperduto PW , Mesko S , Li J , et al. Survival in patients with brain metastases: summary report on the updated diagnosis‐specific graded prognostic assessment and definition of the eligibility quotient. J Clin Oncol. 2020;38:3773‐3784. 32931399 10.1200/JCO.20.01255 PMC7655019

[15] Gaspar L , Scott C , Rotman M , et al. Recursive partitioning analysis (RPA) of prognostic factors in three radiation therapy oncology group (RTOG) brain metastases trials. Int J Radiat Oncol Biol Phys. 1997;37:745‐751. 9128946 10.1016/s0360-3016(96)00619-0

[16] Weltman E , Salvajoli JV , Brandt RA , et al. Radiosurgery for brain metastases: a score index for predicting prognosis. Int J Radiat Oncol Biol Phys. 2000;46:1155‐1161. 10725626 10.1016/s0360-3016(99)00549-0

[17] Nieder C , Mehta MP . Prognostic indices for brain metastases‐‐usefulness and challenges. Radiation Oncology (London, England). 2009;4:10. 19261187 10.1186/1748-717X-4-10 PMC2666747

[18] Nicosia L , Di Muzio J , Agolli L , et al. What is the role of reirradiation in the management of locoregionally relapsed non small‐cell lung cancer? Lung Cancer. 2020;146:263‐275. 32593916 10.1016/j.lungcan.2020.06.017

[19] Chidambaram S , Pannullo SC , Schwartz TH , Wernicke AG . Reirradiation of recurrent brain metastases: where do we stand? World Neurosurg. 2019;125:156‐163. 30738931 10.1016/j.wneu.2019.01.182

[20] Sperduto PW , Kased N , Roberge D , et al. Effect of tumor subtype on survival and the graded prognostic assessment for patients with breast cancer and brain metastases. Int J Radiat Oncol Biol Phys. 2012;82:2111‐2117. 21497451 10.1016/j.ijrobp.2011.02.027 PMC3172400

[21] Sperduto PW , Yang TJ , Beal K , et al. Estimating survival in patients with lung cancer and brain metastases: An update of the graded prognostic assessment for lung cancer using molecular markers (lung‐molGPA). JAMA Oncol. 2017;3:827‐831. 27892978 10.1001/jamaoncol.2016.3834 PMC5824323

[22] Sperduto PW , Jiang W , Brown PD , et al. Estimating survival in melanoma patients with brain metastases: An update of the graded prognostic assessment for melanoma using molecular markers (melanoma‐molGPA). Int J Radiat Oncol Biol Phys. 2017;99:812‐816. 29063850 10.1016/j.ijrobp.2017.06.2454 PMC6925529

[23] Noel G , Proudhom MA , Valery CA , et al. Radiosurgery for re‐irradiation of brain metastasis: results in 54 patients. Radiother Oncol. 2001;60:61‐67. 11410305 10.1016/s0167-8140(01)00359-0

[24] Chao ST , Barnett GH , Vogelbaum MA , et al. Salvage stereotactic radiosurgery effectively treats recurrences from whole‐brain radiation therapy. Cancer. 2008;113:2198‐2204. 18780319 10.1002/cncr.23821

[25] Caballero JA , Sneed PK , Lamborn KR , et al. Prognostic factors for survival in patients treated with stereotactic radiosurgery for recurrent brain metastases after prior whole brain radiotherapy. Int J Radiat Oncol Biol Phys. 2012;83:303‐309. 22079723 10.1016/j.ijrobp.2011.06.1987

[26] Maranzano E , Trippa F , Casale M , et al. Reirradiation of brain metastases with radiosurgery. Radiother Oncol. 2012;102:192‐197. 21880387 10.1016/j.radonc.2011.07.018

[27] Huang Z , Sun B , Shen G , et al. Brain metastasis reirradiation in patients with advanced breast cancer. J Radiat Res. 2017;58:142‐148. 27707842 10.1093/jrr/rrw087 PMC5321192

[28] Cooper JS , Steinfeld AD , Lerch IA . Cerebral metastases: value of reirradiation in selected patients. Radiology. 1990;174:883‐885. 2305074 10.1148/radiology.174.3.2305074

[29] Wong WW , Schild SE , Sawyer TE , Shaw EG . Analysis of outcome in patients reirradiated for brain metastases. Int J Radiat Oncol Biol Phys. 1996;34:585‐590. 8621282 10.1016/0360-3016(95)02156-6

[30] Sadikov E , Bezjak A , Yi QL , et al. Value of whole brain re‐irradiation for brain metastases‐‐single Centre experience. Clin Oncol (R Coll Radiol). 2007;19:532‐538. 17662582 10.1016/j.clon.2007.06.001

[31] Akiba T , Kunieda E , Kogawa A , Komatsu T , Tamai Y , Ohizumi Y . Re‐irradiation for metastatic brain tumors with whole‐brain radiotherapy. Jpn J Clin Oncol. 2012;42:264‐269. 22323551 10.1093/jjco/hys007

[32] Son CH , Jimenez R , Niemierko A , Loeffler JS , Oh KS , Shih HA . Outcomes after whole brain reirradiation in patients with brain metastases. Int J Radiat Oncol Biol Phys. 2012;82:e167‐e172. 21620583 10.1016/j.ijrobp.2011.03.020

[33] Ozgen Z , Atasoy BM , Kefeli AU , Seker A , Dane F , Abacioglu U . The benefit of whole brain reirradiation in patients with multiple brain metastases. Radiat Oncol (London, England). 2013;8:186. 10.1186/1748-717X-8-186 PMC3726464 23879889

[34] Scharp M , Hauswald H , Bischof M , Debus J , Combs SE . Re‐irradiation in the treatment of patients with cerebral metastases of solid tumors: retrospective analysis. Radiat Oncol (London, England). 2014;9:4. 10.1186/1748-717X-9-4 PMC3904456 24387239

[35] Aktan M , Koc M , Kanyilmaz G , Tezcan Y . Outcomes of reirradiation in the treatment of patients with multiple brain metastases of solid tumors: a retrospective analysis. Ann Transl Med. 2015;3:325. 26734635 10.3978/j.issn.2305-5839.2015.12.21 PMC4690995

[36] Logie N , Jimenez RB , Pulenzas N , et al. Estimating prognosis at the time of repeat whole brain radiation therapy for multiple brain metastases: the reirradiation score. Adv Radiat Oncol. 2017;2:381‐390. 29114606 10.1016/j.adro.2017.05.010 PMC5605302

[37] Shaw E , Scott C , Souhami L , et al. Single dose radiosurgical treatment of recurrent previously irradiated primary brain tumors and brain metastases: final report of RTOG protocol 90‐05. Int J Radiat Oncol Biol Phys. 2000;47:291‐298. 10802351 10.1016/s0360-3016(99)00507-6

[38] Rastogi K , Bhaskar S , Gupta S , Jain S , Singh D , Kumar P . Palliation of brain metastases: analysis of prognostic factors affecting overall survival. Indian J Palliat Care. 2018;24:308‐312. 30111944 10.4103/IJPC.IJPC_1_18 PMC6069611

[39] Karam I , Nichol A , Woods R , Tyldesley S . Population‐based outcomes after whole brain radiotherapy and re‐irradiation in patients with metastatic breast cancer in the trastuzumab era. Radiat Oncol (London, England). 2011;6:181. 10.1186/1748-717X-6-181 PMC3259081 22204610

[40] Bernhardt D , Bozorgmehr F , Adeberg S , et al. Outcome in patients with small cell lung cancer re‐irradiated for brain metastases after prior prophylactic cranial irradiation. Lung Cancer. 2016;101:76‐81. 27794411 10.1016/j.lungcan.2016.09.010

[41] Rolfo C , Mack PC , Scagliotti GV , et al. Liquid biopsy for advanced non‐small cell lung cancer (NSCLC): a statement paper from the IASLC. J Thorac Oncol. 2018;13:1248‐1268. 29885479 10.1016/j.jtho.2018.05.030

[42] Raman S , Mou B , Hsu F , et al. Whole brain radiotherapy versus stereotactic radiosurgery in poor‐prognosis patients with one to 10 brain metastases: a randomised feasibility study. Clin Oncol (R Coll Radiol). 2020;32:442‐451. 32085923 10.1016/j.clon.2020.02.001

[43] Holub K , Louvel G . Efficacy of salvage stereotactic radiotherapy (SRT) for locally recurrent brain metastases after initial SRT and characteristics of target population. Clin Transl Oncol. 2021;23:1463‐1473. 33464481 10.1007/s12094-020-02544-y

[44] Minniti G , Esposito V , Clarke E , et al. Stereotactic radiosurgery in elderly patients with brain metastases. J Neurooncol. 2013;111:319‐325. 23187817 10.1007/s11060-012-1016-z

[45] Hazuka MB , Kinzie JJ . Brain metastases: results and effects of re‐irradiation. Int J Radiat Oncol Biol Phys. 1988;15:433‐437. 2841266 10.1016/s0360-3016(98)90026-8

